# Analgesic Effects of* Cnidium officinale* Extracts on Postoperative, Neuropathic, and Menopausal Pain in Rat Models

**DOI:** 10.1155/2019/9698727

**Published:** 2019-06-16

**Authors:** Eun Yeong Lim, Jae Goo Kim, Jaekwang Lee, Changho Lee, Jaewon Shim, Yun Tai Kim

**Affiliations:** ^1^Division of Functional Food Research, Korea Food Research Institute, 245, Nongsaengmyeong-ro, Iseo-myeon, Wanju-gun, Jeollabuk-do 55365, Republic of Korea; ^2^Department of Food Biotechnology, Korea University of Science & Technology, 217 Gajeong-ro, Yuseong-gu, Daejeon 34113, Republic of Korea

## Abstract

*Cnidium officinale*, widely cultivated in East Asia, has been reported to exhibit pharmacological efficacy in various disorders. However, little has been reported on its role as a pain killer. In this study, we reveal that the* C. officinale* extract (COE) has great efficacy as a novel analgesic in various* in vivo* pain models. Administration of COE attenuated hypersensitivity in all postoperative, neuropathic, and menopausal pain models. Decreased hyperalgesia was confirmed by a mechanical withdrawal threshold assay and ultrasonic vocalization call analysis. In addition, application of COE inhibited the induction of the proinflammatory cytokines and calpain-3 on dorsal root ganglion neurons in a spared nerve injury rat model. Treatment with ferulic acid, which was identified as one of the components of COE by HPLC analysis, alleviated nociceptive behaviors. Our findings suggest that ferulic acid is an active compound from COE, and COE is a potential phytomedical source for pain relief by inhibiting the process of inflammation.

## 1. Introduction

Pain is a basic sense essential to the survival of all living things [1]. Pain can signal the need to escape from dangerous stimuli and serve to minimize damage. Despite these benefits, unmanageable and long-lasting pain is one of the principal causes of poor quality of life, which is why many researchers are studying the mechanisms and causes of pain and are looking for novel materials to lessen pain. Although analgesic drugs are currently available and efficient for pain reduction, their repetitive application can result in several side effects, such as physical dependence and tolerance [2, 3]. Several studies also reported that nonsteroidal anti-inflammatory drugs (NSAIDs) might cause gastrointestinal lesions or renal and liver failure [4, 5]. Therefore, there is an emerging need for the discovery of novel materials for producing effective and safe analgesics [6, 7]. Many recent studies have evaluated innovative medical materials based on natural products [8–10].

*Cnidium officinale*, called “Chunkung,” is a flowering plant widely cultivated in Korea, China, and Japan.* C. officinale* is traditionally used in Korea to attenuate pain and increase stamina [11]. Several studies have revealed its beneficial effects, including its antioxidant, anti-inflammatory, anticancer, antiangiogenic, and neuronal cell survival effects, in diverse health problems [12–16]. In one study,* C. officinale *extracts (COE) inhibited an LPS-induced increase in TNF-alpha and IL-12 in BV2 cells [12]. However, little is known of COE's ability to alleviate pain.

Therefore, in this study, we investigated the pain-relieving efficacy of COE in various* in vivo* pain models: a postoperative pain model [17], a neuropathic pain model [18], and a menopausal pain model [19]. In addition, we attempted to determine whether application of COE could be related to the expression of proinflammatory cytokines in a neuropathic pain model. We also showed that ferulic acid, an active compound of COE, could attenuate hyperalgesia.

## 2. Materials and Methods

### 2.1. Experimental Animals

All animal experiments complied with the guidelines of the Korea Food Research Institutional Animal Care and Use Committee (KFRI-M-13003-1). Male Sprague-Dawley (SD) rats were housed, two rats per cage, under a controlled temperature (23°C) and a 12-hour light/dark cycle (Samtako Bio Korea, Gyeonggi-do, Korea). The rats were housed for 1 week for acclimation before the experiments and were anesthetized with 2% isoflurane for the surgery.

### 2.2. Plantar Incision (PI) for Postoperative Pain Rat Model

PI surgery was performed as previously described [17]. Briefly, after rats were anesthetized with 2% isoflurane, a longitudinal incision of approximately 1 cm was performed with a scalpel, through the skin and fascia of the plantar aspect of the paw, starting 0.5 cm from the proximal edge of the heel and extending toward the toes. The plantar muscle was elevated and incised longitudinally. After hemostasis with gentle pressure, the skin was opposed with two single interrupted sutures using polyamide monofilaments. The animals were allowed to recover in a recovery chamber and their home cages. Rats were divided into five treatment groups after PI: (1) PI + vehicle, (2) PI + naproxen (30 mg/kg), (3) PI + COE (30 mg/kg), (4) PI + COE (100 mg/kg), and (5) PI + COE (300 mg/kg). COE was dissolved in distilled water. COE was given per os (p.o.) immediately after PI.

### 2.3. Spared Nerve Injury (SNI) Rat Model of Neuropathic Pain

SNI was performed as previously described [20]. The procedure consisted of an axotomy and ligation of the tibial and common peroneal nerves, leaving the sural nerve intact. The common peroneal and tibial nerves were ligated tightly with 5.0 silk and sectioned distal to the ligation, removing 2~4 mm of the distal nerve stump. Great care was taken to avoid any contact with or stretching of the intact sural nerve. The skin was opposed with two single interrupted sutures using polyamide monofilaments. The sciatic nerve and its branches were identically exposed in the sham control rat group, but they were neither transected nor ligated. Rats were divided into five treatment groups after the SNI surgery: (1) SNI + vehicle, (2) SNI + naproxen (30 mg/kg), (3) SNI + COE (30 mg/kg), (4) SNI + COE (100 mg/kg), and (5) SNI + COE (300 mg/kg). COE was also administered once daily p.o. immediately following surgery, and treatment was continued for 15 consecutive days.

### 2.4. Ovariectomy (OVX) Rat Model of Menopausal Pain

Eight-week-old female SD rats were purchased from Samtako Bio Korea (Gyeonggi-do, Korea). After acclimation for 1 week, rats were anesthetized using 2% isoflurane. Ovaries were removed bilaterally. The ovaries of rats in the sham group were merely touched using forceps. After recovery for 1 week, rats were divided into the following treatment groups: (1) sham + vehicle, (2) OVX + vehicle, (3) OVX + 17*β*-estradiol (E2, 10 *μ*g/kg once daily, i.p), (4) OVX + COE 30 mg/kg, (5) OVX + COE 100 mg/kg, and (6) OVX + COE 300 mg/kg. COE was dissolved in distilled water for oral administration at the desired doses in a volume of 2 mL/kg once daily. E2 was dissolved in distilled water with 1% DMSO and 0.1% Tween 20. All groups were treated for 8 weeks.

### 2.5. Ultrasonic Vocalization (USV) Analysis

Pain-induced USV analysis was performed as previously described [21]. Adult rats were monitored for 22–27 kHz USV emissions after PI, and USVs were scored for 10 min using Sonotrack ultrasonic microphones (Metris B.V., KA, Hoofddorp, Netherlands) placed 25–30 cm from the heads of the animals. The emitted ‘calls' were counted using Sonotrack 2.2.1 software.

### 2.6. Mechanical Withdrawal Threshold (MWT) Analysis

Animals were placed on an elevated wire grid, and the plantar surface of the paw was stimulated using a series of von Frey monofilaments of ascending force (Stoelting, Wood Dale, IL, USA). The MWT was the lowest force that evoked a brisk withdrawal response to one of three repetitive stimuli. A baseline measurement was obtained prior to surgery and at 6 and 24 h after surgery for PI and 3, 6, 9, 12, and 15 days after surgery for SNI to determine the time course of mechanical hyperalgesia.

### 2.7. Cytokine Analysis

All cytokine (interferon (IFN)-*γ*, interleukin (IL)-6, IL-12, and IL-2) levels in isolated L4, L5, and L6 dorsal root ganglion (DRG) of SNI rats were measured using a multiplex ELISA cytokine assay according to the manufacturer's instructions (Quansys Biosciences, Logan, UT, USA).

### 2.8. Immunohistochemistry

DRG were extracted 3 days after SNI and postfixed overnight in 4% paraformaldehyde. Immunofluorescent staining was performed on cryosections (20 *μ*m). Sections were incubated in a solution containing 0.3% Triton X-100 and 3% goat serum for 1 hour at 25°C. Sections were incubated with a calpain-3 antibody (1:200, Sigma, St. Louis, MO, USA) overnight at 4°C. Sections were rinsed five times and incubated with a secondary antibody (Alexa Fluor 488 goat anti-mouse IgG, 1:500, Invitrogen, Carlsbad, CA, USA) for 1 h at 25°C. Sections were mounted in antifade medium and stored at 4°C.

### 2.9. HPLC Conditions

Analytical HPLC analysis was performed using a Jasco HPLC system (Jasco, Hachioji, Tokyo, Japan), which comprised a PU-980 pump and an AS-950-10 autosampler equipped with an MD-2010 Plus multiwavelength detector. Chromatographic separation was performed using a Waters XTerra® RP18 (4.6 mm × 150 mm, particle size 3.5 *μ*m) column. Ultraviolet (UV) detection was measured at 280 nm. A reverse-phase HPLC assay was performed using a mixture of 0.1% acetic acid (A) and 95% methanol in 0.1% acetic acid (B) as the mobile phase. Samples were eluted using the following gradient: 80% A and 20% B as initial conditions, 45% A and 55% B for 6 min, 15% A and 85% B for 5 min, and 80% A and 20% B for 5 min. The flow rate was 1 mL/min, and the column was maintained at 35°C. UV detection was measured at 280 nm. The injection volume was 10 *μ*l of solution. The standard calibration curves of chlorogenic and ferulic acids exhibited linearity (r^2^ > 0.999*∗∗*) in the range of 3.125~50 *μ*g/ml. The concentrations of chlorogenic acid and ferulic acid were 18.176 and 8.805 mg/g, respectively.

### 2.10. Statistical Analysis

Data were analyzed using one-way analysis of variance (ANOVA), followed by Tukey's post hoc test, using Prism 5 (GraphPad Software, Inc., San Diego, CA, USA) for multigroup comparisons. All data are presented as the mean standard error mean (SEM). *∗*, *∗∗*, and *∗∗∗* indicate* p* < 0.05,* p* < 0.01, and* p* < 0.001, respectively.

## 3. Results and Discussion

### 3.1. Mechanical Hypersensitivity Was Reduced by Oral Application of COE in Postoperative Pain Model

**Figure 1:**
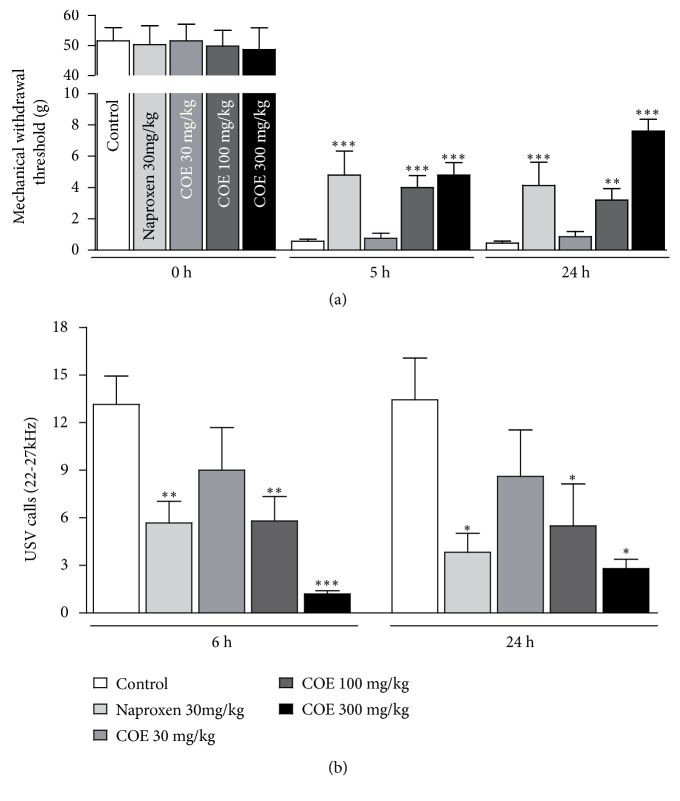
Reduced mechanical sensitivity by application of* C. officinale *extracts (COE) in a postoperative pain model. (a) Compared with the nontreated group, the groups treated with COE (100, 300 mg/kg) showed significantly higher mechanical withdrawal threshold at both 5 and 24 hours after surgery. Naproxen (30 mg/kg) was used as the positive control analgesic. (b) The quantification of 22–27 kHz USV calls revealed a significant difference between the COE-treated and the control groups. All data are the means ± SEM (n = 9 per group). The asterisks indicate significant difference from the control group, *∗* * p* < 0.05, *∗∗* * p* < 0.01, and *∗∗∗* * p* < 0.001.

To assess the efficacy of COE in pain relief, we performed pain-evaluating behavioral tests in postoperative pain rat models. Postoperative pain was induced by a PI to observe the effective reduction of pain. After a longitudinal incision, approximately 1 cm in length, had been made with a scalpel, von Frey experiments were performed independently nine times. Naproxen treatment was used as a pain-reducing control drug through intraperitoneal injection. MWT were measured at 0, 5, and 24 hours after the PI. In the control group (rats without pre- and postoperative treatment of COE), the threshold declined rapidly from 51.500 ± 4.439 (g) to 0.560 ± 0.132 (g) and 0.457 ± 0.120 (g) at 5 and 24 hours after the incision, respectively, indicating that postoperative pain resulting from the PI sufficiently confirmed the pain response. In the group of rats to which 100 mg/kg COE was administered, the threshold reached 4.000 ± 0.777 (g) and 3.200 ± 0.730 (g), at 5 and 24 hours after the incision, respectively. These were significantly higher in the COE-treated group than in the control group at both 5 and 24 hours after incision. A higher threshold value indicates a reduction in hyperalgesia, and the above result suggests that COE relieves postoperative pain. To obtain a more convincing result about the pain-relieving effect of COE, we performed the MWT assay in the 300 mg/kg COE-treated group. As expected, pain relief was found to be COE dose dependent; thresholds reached 4.800 ± 0.800 (g) and 7.600 ± 0.748 (g) at 5 and 24 hours after the incision, respectively (*p *< 0.05, Figure 1(a), Table 1). However, treatment with 30 mg/kg COE did not seem to be enough to reduce hyperalgesia (0.752 ± 0.322 (g) and 0.864 ± 0.340 (g) at 5 and 24 hours after the incision, respectively).

Measuring the USV call is valuable for evaluating emotional status in rodents, as the USV call at 22 to 27 kHz usually occurs when the animals feel pain. We assessed the USV calls of rats after PI to confirm the ameliorative effect of COE. Oral administration of COE after PI reduced USV calls. The number of USV calls in the control group was 13.143 ± 1.792 at 6 hours and 13.429 ± 2.635 at 24 hours after incision, while those in the 100 mg/kg COE-treated group remained at 5.800 ± 1.533 at 6 hours and 5.500 ± 2.634 at 24 hours after incision (Figure 1(b)). An obvious pattern of reduction in USV calls was also detected in the 300 mg/kg treated group (1.200 ± 0.200 at 6 hours, 2.800 ± 0.583 at 24 hours after incision). As in the MWT assay, statistical significance was not observed between the 30 mg/kg COE-treated and the control groups.

### 3.2. Oral Application of COE Attenuated Neuropathic Pain by Inhibiting the Expression of Proinflammatory Cytokines and Calpain-3 in DRG Neurons

**Figure 2:**
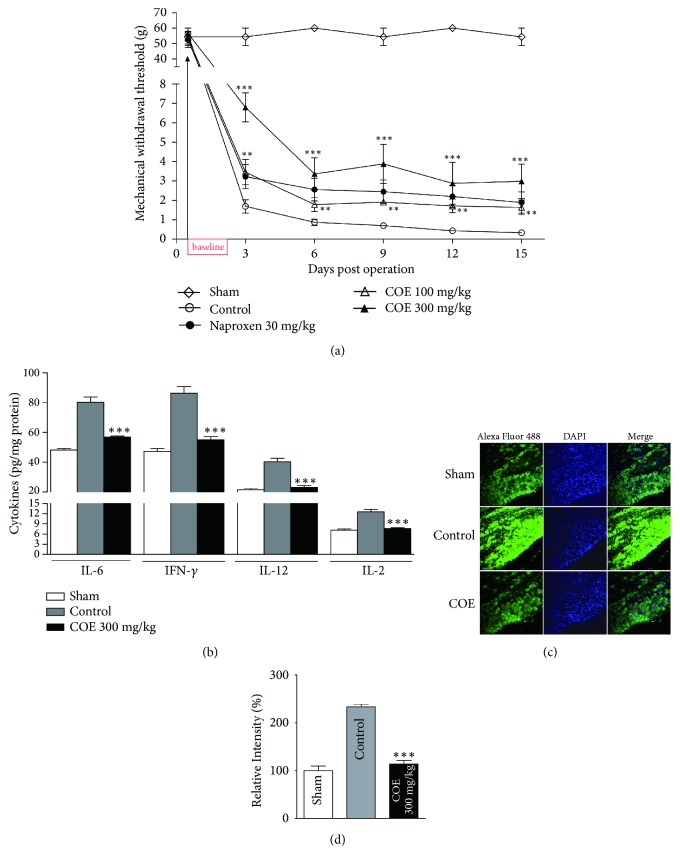
The effect of COE in the SNI neuropathic pain model. (a) Oral administration of COE significantly attenuated hypersensitivity in response to von Frey stimulation from 3 to 15 days in an SNI neuropathic model. The MWTs of the group treated with COE or naproxen remained higher than those of the untreated control group. (b) Administration of COE significantly inhibited the induction of IL-6, IFN-*γ*, IL-12, and IL-2 expression in DRG neurons of a neuropathic rat model by ELISA. Representative images (c) and relative fluorescence intensity (d) against calpain-3 (Alexa Fluor 488) revealed that COE attenuated the expression of calpain-3. Data are the means ± SEM (n = 9 per group). The asterisks indicate significant difference from the control group, *∗∗* * p* < 0.01 and *∗∗∗* * p* < 0.001.

We assessed whether COE could ameliorate neuropathic pain in addition to postoperative pain. To induce neuropathic pain, we performed a SNI. SNI is a canonical method for causing neuropathic pain because it exhibits high reproducibility. The SNI procedure consists of ligation of the tibial and peroneal nerves after axotomy. We then evaluated MWT through von Frey assays every 3 days after the procedure. In the sham group, in which the sciatic nerves were exposed but neither transected nor ligated, MWT remained almost unchanged (Figure 2(a)). However, the control rats showed severe susceptibility 3 days after surgery. As in the postoperative pain experiment, naproxen was used as the positive control drug for pain reduction. The threshold scores were significantly higher in the groups treated with naproxen or COE than in the groups without any treatment (control), indicating that rats treated with COE were less susceptible to neuropathic pain than control rats (Figure 2(a)). These results show that COE is effective in a neuropathic pain model as well as in a postoperative pain model.

Inflammation is believed to be involved in the process of pain [10, 22]. Pain is associated with tissue injury and inflammation [22, 23] and is a characteristic symptom of arthritis [24]. Inflammatory mediators contain cytokines released from injured tissue to activate and sensitize nerve terminals. Recent studies have reported that not only immune cells but also DRG neurons release proinflammatory cytokines [25]. It has been reported that proinflammatory IL-1*β* expression is enhanced by crush injuries to peripheral nerves and causes increased levels of substance P and prostaglandin E2 in neuronal cells [26]. To elucidate the efficacy of COE in inflammation, we measured the expression levels of representative proinflammatory cytokines, IL-6, IFN-*γ*, IL-12, and IL-2 from DRG neurons after SNI. At 15 days after SNI, the expression levels of cytokines (IL-6: 80.195 ± 3.570, IFN-*γ*: 86.390 ± 4.376, IL-12: 40.391 ± 2.224, and IL-2: 12.554 ± 0.716) increased significantly compared with those after the sham operation (IL-6: 48.214 ± 0.999, IFN-*γ*: 47.298 ± 1.853, IL-12: 21.406 ± 1.017, and IL-2: 7.156 ± 0.337). Administration of COE (300 mg/kg) before and after the procedure resulted in a decrease of proinflammatory cytokine expression levels in DRG neurons, despite SNI (IL-6: 56.917 ± 0.661, IFN-*γ*: 55.189 ± 1.927, IL-12: 23.157 ± 1.166, and IL-2: 7.623 ± 0.248) (Figure 2(b)). Therefore, it is thought that application of COE shows an analgesic effect by reducing the expression levels of cytokines and inhibiting the inflammatory cascade. Despite this finding, further studies are needed to define the active and effectual components in COE and the exact mechanism on how it inhibits the expression of proinflammatory cytokines.

To find genes associated with neuropathic pain on DRG neurons, we performed a microarray analysis comparing the DRG of SNI in sham groups. Calpain-3 is one of the genes upregulated by neuropathic pain (data not shown). It is a member of the calpain family of calcium-dependent intracellular proteases. Its mRNA levels are high in the muscle and it is known to be involved in muscle regeneration [27, 28]. The relationship of calpain-3 and pain was not investigated previously, and the present study is the first to suggest an association of calpain-3 with neuropathic pain. Well-known calpain family proteins are calpain-1 and -2, which are associated with neuropathic pain [29, 30]. We confirmed the increase in calpain-3 in the SNI model by immunostaining with calpain-3 antibody in L5 DRG sections. Oral administration of COE significantly decreased calpain-3 upregulation. The relative intensity of fluorescence of calpain-3 increased in the control group (233.633 ± 5.229) compared to that in the sham group (100.000 ± 9.662), which was consistent with the microarray data. This increase disappeared in the group treated with 300 mg/kg COE (114.067 ± 7.358) (Figures 2(c) and 2(d)). This result indicated that COE attenuated neuropathic pain by inhibiting the expression of calpain-3.

### 3.3. Mechanical Hypersensitivity Was Reduced by Oral Application of COE in Menopausal Pain Model

**Figure 3:**
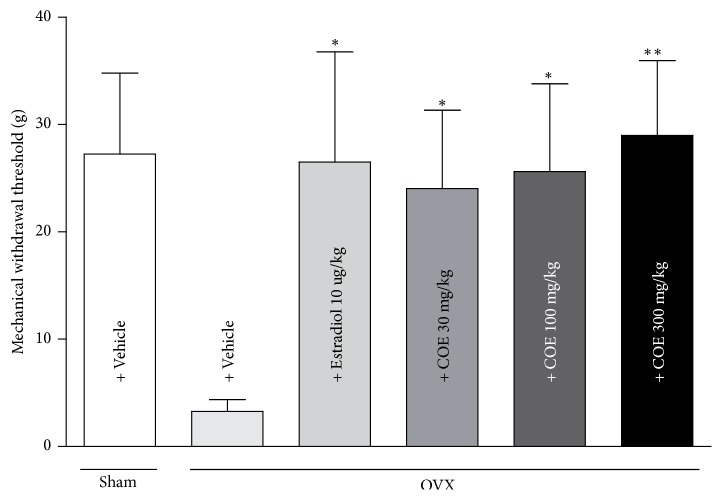
Reduced mechanical sensitivity by application of COE in OVX rats. COE alleviated pain sensitivity in menopausal pain model. Data are the means ± SEM (n = 9 per group). The asterisks indicate significant difference from the control group, *∗* * p* < 0.05 and *∗∗* * p* < 0.01.

The above-mentioned experiments confirmed the efficacy of COE in postoperative and neuropathic pain. An OVX model was used to examine its effect in menopausal pain [19]. We evaluated whether COE attenuated OVX-induced pain sensitivity. Oral application of COE reduced menopausal pain sensitivity in a concentration-dependent manner (24.04 ± 7.303 g at 30 mg/kg, 25.63 ± 8.187 g at 100 mg/kg, and 29.00 ± 6.974 g at 300 mg/kg) (Figure 3). Menopause occurs when the amount of estrogen released from the ovaries decreases as a woman ages, and it may cause pain sensitivity [31]. Pain sensitivity increases in the abdomen, hind limbs, and proximal tail of OVX rats, and these symptoms are alleviated following hormone administration [32]. Estradiol administration reversed OVX-induced hypersensitivity.

### 3.4. The Active Compound of COE, Ferulic Acid, Attenuated Postoperative Pain

**Figure 4:**
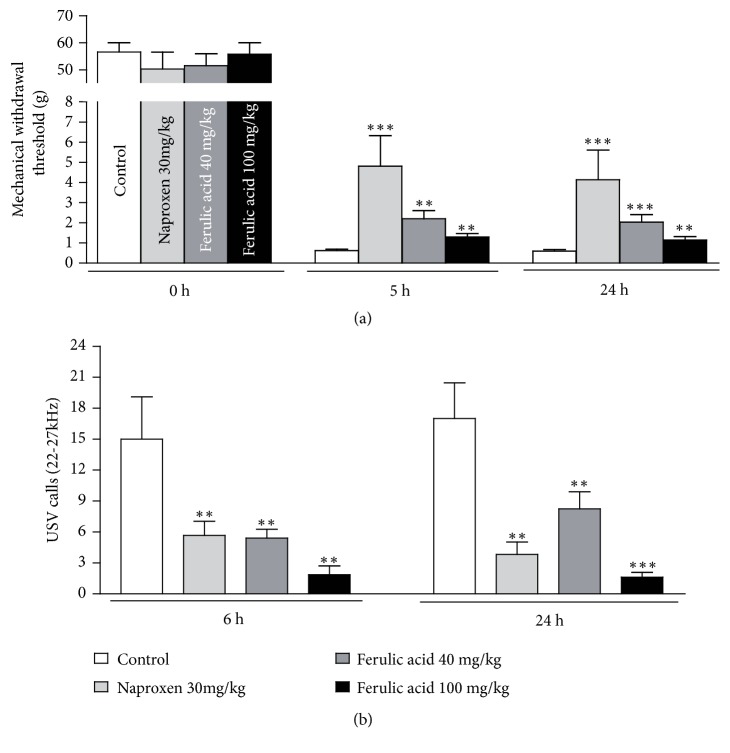
Reduced pain sensitivity by application of ferulic acid in a postoperative pain model. (a) Compared with the nontreated group, the group treated with ferulic acid showed a significantly higher mechanical withdrawal threshold (a) and less USV calls (b) at both 5 and 24 hours after surgery in a dose-dependent manner. Naproxen (30 mg/kg, n = 9) was used as the positive control analgesic. Data are the means ± SEM (n = 9 per group). The asterisks indicate significant difference from the control group, *∗∗* * p* < 0.01 and *∗∗∗* * p* < 0.001.

To identify the components of COE that relieved pain, HPLC was performed and ferulic acid was isolated. It was orally administered after induction of postoperative pain. The significant development of mechanical allodynia was confirmed by a decrease in the withdrawal threshold values (56.60 ± 3.400 at 0 h to 0.600 ± 0.073 at 24 h after PI). The MWTs of the ipsilateral hind paw 24 h after surgery demonstrated that a single ferulic acid treatment (40 to 100 mg/kg) attenuated mechanical allodynia (2.033 ± 0.366 at 40 mg/kg and 1.350 ± 0.155 at 100 mg/kg). Similar effects were observed for naproxen (30 mg/kg) administration, which was used as a positive control (4.133 ± 1.481 g) (Figure 4(a)). USV calls (22-27 kHz) in the ferulic acid-treated group (8.250 ± 1.652 calls at 40 mg/kg and 1.625 ± 0.460 calls at 100 mg/kg) also decreased compared to those in the control group (17.000 ± 3.464 calls) 24 hours after PI (Figure 4(b)). The major components of COE include ferulic acid, senkyunolide I, senkyunolide H, senkyunolide A, Z-ligustilide, and levistolide A [33]. Based on this, we could detect chlorogenic acid, ferulic acid, senkyunolide A, and Z-ligustilide from COE using HPLC (Table 1). Ferulic acid is one of the most abundant phenolic acids in plants and a major component of COE. It reduces collagen-induced inflammatory cytokine production [34]. Ferulic acid exhibited antinociceptive effects in a chronic neuropathic pain model induced by constriction injury and also alleviated pain in other models of neuropathic pain, such as the chemotherapy-induced neuropathy model [35]. Although ferulic acid has been reported to aid in alleviating chronic pain, this is the first study showing that ferulic acid could help alleviate postoperative pain.

## 4. Conclusions

In this study, we demonstrated that COE has great analgesic efficacy. Oral administration of COE attenuated hypersensitivity in* in vivo *models of postoperative, neuropathic, and menopausal pain. In addition, application of COE inhibited the induction of the proinflammatory cytokines and calpain-3, which was induced by neuropathic pain on DRG neurons. Ferulic acid was expected to be a potent active compound of COE for pain relief from HPLC analysis. Ferulic acid effectually alleviated mechanical hypersensitivity in a postoperative pain model. Although further studies about the exact working mechanism of COE in pain relief are required, COE might be a novel natural product for reducing hypersensitivity in postoperative, neuropathic, and menopausal pain.

## Figures and Tables

**Table 1:** Chemical compounds content and retention time of COE.

Compound	RT (min)	Content (%)
Chlorogenic acid	6.943	1.8176
Ferulic acid	7.945	0.8805
Senkyunolide A	13.152	1.1817
(Z)-Ligustilide	14.445	1.1775

## Data Availability

The data used to support the findings of this study are included within the article.
